# Measuring Achievement, Affiliation, and Power Motives in Mobility Situations: Development of the Multi-Motive Grid Mobility

**DOI:** 10.3389/fpsyg.2021.765627

**Published:** 2022-01-03

**Authors:** Alica Mertens, Maximilian Theisen, Joachim Funke

**Affiliations:** Institute of Psychology, Heidelberg University, Heidelberg, Germany

**Keywords:** mobility situations, psychological motives, grid technique, age, physical constraint

## Abstract

The current study introduces the Multi-Motive Grid Mobility (MMG-M) in an age-stratified sample (*N* = 206) that aims to disentangle six motive components – hope of success, hope of affiliation, hope of power, fear of failure, fear of rejection, and fear of power – in mobility-related and mobility-unrelated scenarios. Similar to the classical Multi-Motive Grid (MMG), we selected 14 picture scenarios representing seven mobility and seven non-mobility situations. The scenarios were combined with 12 statements from the MMG. Both the MMG-M and MMG were assessed to allow comparability between psychometric criteria. The results of confirmatory factor analyses yielded a good model fit for a six-factor solution with an additional mobility factor for the MMG-M. Internal consistency of the items was similar to the MMG. Lastly, we investigated associations between the motive components and mobility-related variables. We found that risk awareness was positively related to all fear components in both mobility and non-mobility scenarios. Most importantly, physical constraint was positively associated with fear of rejection and fear of power in mobility situations underlining the importance to create support systems to reduce these concerns in people’s everyday lives.

## Introduction

Maintaining high mobility in life is crucial as it allows individuals to lead independent and dynamic lives, which is essential to preserve high levels of social participation (Webber et al., 2010). The loss of mobility has been found to be associated with various psychological dimensions, like well-being (Stanley et al., 2011), quality of life (Metz, 2000; Groessl et al., 2007; Yeom et al., 2008), depressive symptoms (Hirvensalo et al., 2007), or mortality (Bergland et al., 2017). These empirical findings underline the importance of mobility, especially in an aging society. Thus, it is not surprising that there is a growing interest in identifying factors that influence mobility (Webber et al., 2010).

While the mobility concept is often used more or less simultaneously with travel and physical movement (Metz, 2000), an increasing number of researchers seek to discover mobility determinants that go beyond these two aspects, for example cognitive, or psychosocial factors (Webber et al., 2010). While there is already an existing body of research investigating the interplay between cognitive domains (e.g., memory, problem-solving, and attention) and mobility (Demnitz et al., 2017; de Silva et al., 2019; Sunderaraman et al., 2019), or psychosocial factors (e.g., self-efficacy: Perkins et al., 2008; depression: Gayman et al., 2008; social environment: Rudman et al., 2006), to our knowledge, there is no research targeting the motive-related side of mobility, yet. Going beyond the idea of mobility as a physical or transportation-related dimension, we want to create a way to assess fundamental psychological motives related to mobility.

Established motives associated with achievement, affiliation, and power can be measured with the Multi-Motive Grid (MMG; Sokolowski et al., 2000). Additionally, the MMG distinguishes hope (focus on the possibility of success) and fear components (focus on the possibility of failure) for each motive. It is usually assumed that such measured motives are transsituational (they should relate to situations of all kinds). However, it seems plausible that specific motives might be addressed more strongly in mobility-associated situations, especially if people have a certain frailty level which is associated with higher age (see also Hou et al., 2020, for an adaption of the original MMG to Social Networking Use). Therefore, the aim of the current study is to develop a motive grid assessing the three fundamental psychological motives of achievement, affiliation, and power as well as their corresponding hope and fear components in mobility and non-mobility situations.

### The Mobility Concept

The concept of mobility is typically defined as the ability of individuals to move themselves (movement capability) and also includes the movement from one environment to another with some sort of transportation (e.g., Stalvey et al., 1999). The usage of assistive devices in order to enable a specific movement is also included in the mobility concept (Webber et al., 2010). Early work investigating mobility concentrated on the environment and the person-environment fit (Lawton and Nahemow, 1973). More recent studies have also focused particularly on the effects of environmental aspects on mobility (Clarke et al., 2008; Nagel et al., 2008; Penger and Oswald, 2017; Stafford and Baldwin, 2018; Ottoni et al., 2021) and on the question, which transportation forms are essential to maintain older individual’s access to important services, people, and activities (Oxley and Whelan, 2008; Dickerson et al., 2019).

We agree with the definitions of mobility that are the basis of the outlined studies. However, only focusing on the physical and environmental aspects of mobility neglects the social and psychological role that mobility plays for individuals and society (Webber et al., 2010). In their conceptual model, Webber et al. (2010) identified seven life-space locations (room, home, outdoors, neighborhood, service community, surrounding area, and world) that are composed of five essential mobility determinants. These mobility determinants include physical, environmental, cognitive, financial, and psychosocial factors. Psychosocial determinants represent factors such as coping strategies, depression, fear, or self-efficacy beliefs that influence the motivation or the intention to be mobile. Whereas there is already evidence on the association between mobility – or the motivation to be mobile – and self-efficacy (Perkins et al., 2008), depression (Gayman et al., 2008; Rosenberg et al., 2013; Gay et al., 2019), or fear of falling (Maki et al., 1991; Friedman et al., 2002; Litwin et al., 2018), to our knowledge no research has investigated fundamental psychological motives in the context of mobility. Therefore, we would like to enrich the framework of Webber et al. (2010) by investigating the existence of mobility-related motives. Understanding underlying motives that drive a person to engage in or to circumvent mobility situations is essential to gain a more complex picture in order to comprehend mobility decisions in the elderly or in individuals with physical restrictions.

### Psychological Motives and the Grid Technique

In the last century, the investigation of human motivation was of central focus in personality research (e.g., McClelland, 1951; Spence, 1958; Schüler et al., 2008; Müller and Cañal-Bruland, 2020; Schütz and Schultheiss, 2020). The motive disposition theory (MDT; e.g., McClelland, 1987) can be described as interactionist: It assumes that motives are dispositions “to respond to specific classes of target states with typical affect patterns” (Langens et al., 2005, p. 73). Therefore, they are usually assumed to generalize across situations. At the same time, motives are understood as the result of learning experiences. MDT differentiates between three fundamental psychological motives: achievement, affiliation, and power. Individuals with a high achievement motive strive for improving their personal performance and for accomplishing challenging goals. Affiliation oriented individuals seek social interaction with others and put effort into maintaining emotional meaningful relationships. Lastly, people with a stronger power motive have a higher intention to increase their social status and to control or influence others. For each motive, two sub-dimensions exist – the hope and fear component. The hope component describes the focus on the possibility of successfully fulfilling the motive. The fear component describes the focus on the possibility of failing to fulfill the motive. Up to date, there are three different ways to assess these motives and their respective components: self-report measures (e.g., Mehrabian, 1970), projective techniques [e.g., the Thematic Apperception Test (TAT); Murray, 1943; the Picture Story Exercise (PSE); McClelland et al., 1989], and grid techniques [e.g., the Multi-Motive Grid (MMG); Sokolowski et al., 2000]. Whereas self-report measures rather assess the explicit component of the specific motives, which refers to the consciously accessible aspects of the self-concept, projective techniques grasp the more unconscious or implicit part of motive disposition (McClelland et al., 1989).

The MMG represents a semi-projective measure which combines features from the TAT (Murray, 1943) as well as questionnaire measures and has been commonly used for the assessment of implicit motives (e.g., Puca and Schmalt, 1999; Langens and Schmalt, 2002; Nikitin and Freund, 2015). There is, however, some debate on whether the MMG is an implicit (e.g., Langens and Schmalt, 2008) or rather a semi-implicit motive measure (Schüler et al., 2015). Implicit measures have the advantage of measuring motives that participants might not be able or not be willing to reveal in an explicit measure. Even though the MMG is considered to be not purely implicit, we hope that the indirect nature of this measurement allows to capture motives that participants are either not aware of or that they would not be willing to share. The MMG enables assessing all three psychological motives in one single measure simultaneously by combining selected pictures with 12 statements representing binary items that can be agreed or disagreed on (Sokolowski et al., 2000).

In the literature, it is usually assumed that learning experiences generalize across situations to form situation-independent motives. Instead, we argue that the concept of learned motives allows different motive structures to be active in situations with different learning history. Imagine a person that has had a severe mobility limitation for all their life. They might have a high achievement motive in situations where their mobility limitation does not play any role. This motive might have a strong hope component because they are very competent in these situations. However, in mobility-related situations, this person might have a lower power motive because they have had less rewarding experiences in such situations. Instead, they might have learned that it can be highly pleasant to have fulfilling connections to other people in such mobility-associated situation. Therefore, their affiliation motive might become more active in situations of mobility than it would be in non-mobility situations. It might also be possible that individuals who experience mobility impairments in their life either abruptly (e.g., due to an accident) or gradually (through aging) shift their motive focus. Although we also consider a general disposition of certain motives, it is possible that some features of the presented scenarios become more salient (e.g., busy traffic or physical effort) when a person’s mobility is restricted. When measuring motives in a more general way, these differences might not be captured.

### Motives for Mobility and Physical Activity

Recent studies investigating motives related to mobility or physical activity focused on general reasons for being physically active or mobile than on differentiating between fundamental motives in mobility situations (e.g., Rasinaho et al., 2007; Gutiérrez et al., 2018; Rodrigues et al., 2019). Gutiérrez et al. (2018) identified three latent classes that characterized motives to physical exercise in older individuals. One class represented the motive to socialize with others when being physically active and framed physical exercise as a leisure activity. The second class was rather the performance side of physical exercise that allows individuals to stay physically fit whereas the third class represented the motive to exercise for medical advice. Rasinaho et al. (2007) examined motives as well as barriers to mobility among older adults with mobility limitations. Individuals with mobility restrictions more often reported that a lack of company, fear, or negative experiences represent barriers to being mobile compared to older adults without mobility limitations. Rodrigues et al. (2019) found that for older adults, health pressure and revitalization played a major role when deciding to exercise while younger individuals were more interested in increasing physical strength and appearance compared to their older counterparts.

The mentioned findings demonstrate the importance to address a diverse range of motives that underlie physical activity or mobility in older and younger adults. What becomes apparent is that not only fitness or health reasons promote active behavior in the elderly, but that also social factors (e.g., being in company with others when exercising) represent an essential reason for being active. Whereas reasons for physical activity were often the focus of previous investigations, the examination of fundamental psychological motives in mobility situations is missing. Based on the above-mentioned empirical findings, it is reasonable that – for instance – the achievement motive might be more pronounced in mobility situations for individuals whose mobility is restricted because these situations represent a stronger performance-related character for them. Moreover, also the affiliation motive (especially the component “fear of rejection”) might be triggered more strongly for physically restricted individuals in those situations because they fear not being able to keep up with the “fit” ones. Lastly, it is also possible that situations related to mobility set off a feeling of powerlessness for individuals with physical restrictions because of the perception that these demanding situations cannot be managed in the first place. Especially the motive component “fear of power” might be triggered in those kinds of situations. Up until now, no measure exists that is able to capture the three fundamental motives (achievement, affiliation, and power) in mobility situations and compare them to scenarios in which mobility is less of an issue.

### The Current Study

The aim of the current study is to develop a motive assessment that enables measuring the three fundamental psychological motives – achievement, affiliation, and power – in mobility and non-mobility situations. Based on the grid technique by Sokolowski et al. (2000), we created picture scenarios and accompanied them with the 12 statements of the classical MMG that participants could agree or disagree on. The picture scenarios were selected based on whether or not they incorporated the theme “mobility” in their presented scenarios. To address the question whether the presented pictures incorporate the theme “mobility,” we used two approaches. On the one hand, we asked participants directly how much the pictures referred to mobility to them. On the other hand, we used a response time task, in which participants should decide as quickly as possible whether the pictures scenario represented a mobility theme or not. With these two methodological approaches, we intended to capture a direct and indirect assessment of mobility.

We recruited participants of different ages to test the psychometric criteria of the picture scenarios and the associated statements in an age-stratified sample. The classical MMG scenarios were additionally assessed in our study to allow comparing psychometric criteria (e.g., motive structure, internal consistencies, and motive correlations) between the classical MMG and the newly developed Multi-Motive Grid Mobility (MMG-M). We argue that differences in the motive structures between mobility and non-mobility situations might be especially prominent in persons that have made systematically different learning experiences in mobility situations in comparison to non-mobility situations. To validate this reasoning, we also assessed different mobility-related variables (e.g., age, general health, physical constraint, and awareness of risk of physical/health constraint) to investigate associations with motive components in mobility-related and –unrelated scenarios in an exploratory way.

We intentionally selected the MMG technique of Sokolowski et al. (2000) as basis for our MMG-M because it is relatively easy to perform especially for older individuals or people with mobility restriction: participants only have to indicate whether they agree or disagree with the respective 12 statements regarding the presented picture scenarios. In contrast, the Picture-Story Exercise (PSE; McClelland et al., 1989), for example, requires individuals to write imaginative stories which is much harder to perform and analyze compared to the predefined statements of the MMG. Therefore, we perceived the technique developed by Sokolowski et al. (2000) as more appropriate for our research goal. In contrast to a pure self-report measure, the graphical nature of the MMG allows a relatively straightforward adaptation for mobility contexts, whilst preserving the comparability with the original measure.

With the creation of the MMG-M, we hope to achieve a better understanding of underlying psychological motives in mobility-related situations among individuals of different age or frailty levels. Information about differences in fundamental psychological motives in mobility scenarios regarding older people or individuals with mobility limitations might help tailor assistance and support systems that support engagement in or maintenance of physical activity among those groups.

## Materials and Methods

### Participants

To analyze the model fit of the motive structure of the MMG-M using confirmatory factor analysis, a minimum sample size of 200 participants is often suggested in the literature (e.g., Anderson and Gerbing, 1984; Hoogland and Boomsma, 1998; MacCallum et al., 1999; Hoe, 2008). We used an online questionnaire for data collection to reach a large number of participants of different ages. The link to the questionnaire was posted on several platforms and was also distributed by researchers of the “Network Aging Research” department of Heidelberg University. Three-hundred-and-seventy-six participants started the questionnaire, 209 participants (56%) completed the online survey. Three participants were excluded from the analyses, as they responded to all items with the same response alternative. The final sample consisted of 206 participants (*M*_*age*_ = 35.78 years, *SD*_*age*_ = 20.34, range = 18 – 89). Sixty-nine percent indicated to be female, 29% to be male, and 2% stated to be gender diverse. Mean years of education among participants was 16.37 years (*SD* = 4.12). Before participants started with the online survey, they received information about all study details and gave their informed consent. They could either participate for course credit or take part in a lottery to win a voucher of 50€. The study took about 45–60 min.

We additionally recruited participants for a short follow-up survey to investigate the arousal potentials of the pictures for the MMG-M like it was done in previous studies of the classical MMG (Schmalt et al., 1994; Sokolowski et al., 2000). The link to the online survey was sent to those participants who provided their e-mail addresses in the main study. Sixty participants started the online questionnaire, 52 (87%) participants completed the study (*M*_*age*_ = 52.96 years, *SD*_*age*_ = 24.09, range = 20–89). Sixty percent stated to be female, 36% to be male, and 4% indicated to be gender diverse. The participants had an average of 16.70 years of education (*SD* = 3.18). They received information about the study details and provided their informed consent for participation. As an incentive, 1€ per participant was donated to a charitable organization focusing on the needs of elderly people living in poverty. The follow-up survey took about 15 min.

### Measures

#### Multi-Motive Grid Mobility Pictures

**FIGURE 1:**
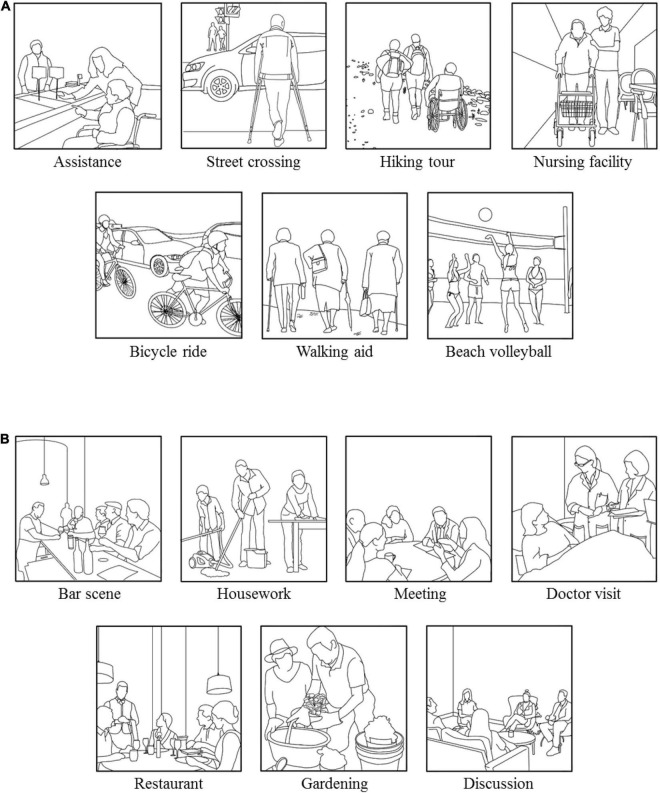
Illustration of the final selection of picture scenarios presenting mobility **(A)** and non-mobility **(B)** situations.

To create our multi-motive grid for mobility and non-mobility situations, we selected picture material from the internet presenting scenarios in which mobility is represented quite strongly and situations in which mobility is less pronounced. We chose a total amount of 28 pictures and created the motive scenarios by drawing these pictures in the style of the typical MMG pictures (Sokolowski et al., 2000). Figure 1 exemplifies the style. As we intended to compare the motive scores between the MMG-M scenarios with those of the classical MMG, the pictures needed to have the same style characteristics in order to prevent that differences in terms of image type influence the activation of motives in the scenarios. To assess the three psychological motives in those situations with their hope and fear components – hope of success (HS), hope of affiliation (HA), hope of power (HP), fear of failure (FF), fear of rejection (FR), and fear of power (FP) – we used the same 12 statements that are implemented in the classical MMG (Sokolowski et al., 2000). This was also done to allow comparability between the psychometric criteria of the MMG-M and the MMG.

From these 28 pictures showing mobility-related and rather mobility-unrelated scenarios, we chose 14 pictures – seven of them showing mobility and seven presenting non-mobility scenarios – following three main criteria: thematic content, coverage of all motives, and arousal potential.

For the first criterion, we asked participants to rate the thematic content of each picture. We asked the following question: “How much does the above situation refer to mobility to you?”. For each picture scenario, we additionally used two items that replaced the word “mobility” with the word “activity” and the word “health,” because we wanted it to be less obvious that the pictures differed in terms of mobility. The mean mobility ratings differed significantly between the selected mobility and non-mobility scenarios, *t*(205) = 31.42, *p* < 0.001, *d* = 2.21, 95% *CI* [2.35, 2.87]. Mean activity and health ratings also differed significantly between the selected mobility and non-mobility scenarios, *t*(205) = 19.65, *p* < 0.001, *d* = 1.37, 95% *CI* [1.66, 2.12] and, *t*(205) = 24.70, *p* < 0.001, *d* = 1.73, 95% *CI* [1.68, 2.14], respectively. However, the difference (mobility versus non-mobility) in mobility ratings was significantly stronger compared to the difference in activity ratings, *t*(205) = 16.02, *p* < 0.001, *d* = 1.12, 95% *CI* [0.84, 1.25], and health ratings, *t*(205) = 13.64, *p* < 0.001, *d* = 0.96, 95% *CI* [0.68, 1.09].

Secondly, we intended that our final selection of picture material should differentiate well between the psychological motives. Thus, we decided to select pictures of which the corresponding statements showed a sufficient range of item difficulty (ideally ranging between 0.20 and 0.80)^1^.

For the last criterion, we used the additional sample (*N* = 52) to compute the arousal potentials of each picture, following the procedure described in Schmalt et al. (1994). This way, it is possible to examine which motives are aroused in the respective picture scenarios and to investigate whether the presented scenarios have different levels of ambiguity. Participants answered on a 5-point Likert-type scale (ranging from *very little* to *very much*) to the following question: “How much does the above situation illustrate an achievement theme?” The two other questions referred to affiliation and power, respectively. The arousal potentials were calculated by *z*-transforming the indicated motive arousals across pictures. Accordingly, a positive value means that the associated motive is prominent in the respective picture.

Using these three criteria, we were able to select seven mobility-related scenarios and seven mobility-unrelated scenarios. The final selection of pictures is shown in Figure 1. The arousal potentials of the selected pictures are displayed in Table 1. While some pictures possess low ambiguity like the “Bar scene,” which arouses only the affiliation motive, the picture scenario “Nursing facility” arouses all three motives simultaneously. Mean mobility estimates for each picture and item difficulties for each statement are depicted in Table 2.

**TABLE 1:** Arousal potentials (z-transformed) for the three fundamental motives and the attached arousal types of the pictures of the Multi-Motive Grid Mobility (MMG-M).

Title of picture	Achievement	Affiliation	Power	Arousal type		
**Mobility**						
(1) Assistance	–0.15	0.12	0.68		Affiliation	Power
(2) Street crossing	0.28	–0.89	0.29	Achievement		Power
(3) Hiking tour	0.78	0.70	–0.07	Achievement	Affiliation	
(4) Nursing facility	0.43	0.28	0.28	Achievement	Affiliation	Power
(5) Bicycle ride	0.04	–0.42	0.37	Achievement		Power
(6) Walking aid	–0.20	0.62	–0.71		Affiliation	
(7) Beach volleyball	0.97	0.94	–0.16	Achievement	Affiliation	
**Non-mobility**						
(8) Bar scene	–1.15	0.76	–0.40		Affiliation	
(9) Housework	0.67	–0.05	–0.22	Achievement		
(10) Meeting	0.77	0.18	1.01	Achievement	Affiliation	Power
(11) Doctor visit	0.25	–0.73	1.41	Achievement		Power
(12) Restaurant	–0.53	0.52	0.22		Affiliation	Power
(13) Gardening	0.02	–0.14	–0.28	Achievement		
(14) Discussion	–0.91	0.73	–0.02		Affiliation	

*N = 52. Positive z-values indicate the presence of a specific arousal type.*

**TABLE 2:** Mean and standard deviation of mobility ratings, mean mobility decisions (MD)*^a,b^*, and item difficulty of each statement for the selected picture scenarios.

Title of picture	Mobility	MD	HS1	HS2	HA1	HA2	HP1	HP2	FF1	FF2	FR1	FR2	FP1	FP2
**Mobility**														
(1) Assistance	4.04 (1.06)	0.57	0.60	0.54	0.74	0.46	0.62	0.50	0.67	0.26	0.47	0.30	0.33	0.45
(2) Street crossing	4.40 (0.88)	0.92	0.67	0.51	0.24	0.23	0.60	0.22	0.69	0.44	0.39	0.15	0.30	0.57
(3) Hiking tour	4.54 (0.78)	0.97	0.81	0.79	0.79	0.60	0.67	0.59	0.69	0.25	0.46	0.37	0.26	0.29
(4) Nursing facility	4.40 (0.90)	0.91	0.74	0.66	0.82	0.46	0.64	0.54	0.63	0.33	0.45	0.42	0.33	0.36
(5) Bicycle ride	4.59 (0.70)	0.97	0.62	0.57	0.43	0.28	0.48	0.30	0.30	0.22	0.24	0.09	0.21	0.49
(6) Walking aid	4.15 (0.96)	0.91	0.54	0.49	0.87	0.62	0.41	0.39	0.42	0.32	0.27	0.27	0.23	0.13
(7) Beach volleyball	4.03 (1.17)	0.84	0.89	0.88	0.85	0.67	0.68	0.81	0.62	0.25	0.47	0.17	0.61	0.40
**Non-mobility**														
(8) Bar scene	2.18 (1.17)	0.13	0.32	0.25	0.86	0.74	0.42	0.62	0.15	0.30	0.57	0.55	0.57	0.20
(9) Housework	2.94 (1.21)	0.56	0.55	0.43	0.41	0.28	0.42	0.38	0.30	0.25	0.21	0.11	0.26	0.19
(10) Meeting	1.97 (1.06)	0.09	0.87	0.81	0.57	0.61	0.87	0.86	0.46	0.22	0.76	0.52	0.73	0.66
(11) Doctor visit	2.80 (1.41)	0.18	0.51	0.40	0.60	0.25	0.69	0.40	0.52	0.28	0.28	0.15	0.34	0.55
(12) Restaurant	2.24 (1.20)	0.10	0.44	0.36	0.79	0.64	0.50	0.67	0.25	0.24	0.43	0.56	0.49	0.27
(13) Gardening	2.56 (1.15)	0.46	0.74	0.81	0.74	0.63	0.53	0.52	0.36	0.12	0.18	0.15	0.15	0.09
(14) Discussion	1.96 (1.02)	0.09	0.58	0.43	0.80	0.67	0.60	0.72	0.26	0.24	0.62	0.65	0.61	0.39

*^a^This variable indicates the percentage of mobility decisions. A value of 1.00 would indicate that all participants decided the scenario to be mobility-related.*

*^b^N = 192. HS, hope of success; HA, hope of affiliation; HP, hope of power; FF, fear of failure; FR, fear of rejection; FP, fear of power.*

#### Physical Constraint

We used six items from the SF-12 (Ware et al., 1996) to assess the physical constraint of participants. Regarding the first two items, participants had to answer the following question: “Are you restricted in the following activities due to your current state of health?” Participants had to indicate whether they felt *strongly* restricted, *slightly* restricted, or *not at all* restricted in *moderately difficult physical activities* (*e.g., move a table, vacuuming, bowling, playing golf, etc.*) and when *climbing several stairs*. For the second two items, the participants answered another question: “In the past 4 weeks, how many times have you had any difficulties at work or other everyday activities at work or at home because of your physical health?” The participants indicated their response on a 5-point Likert-type scale (“never,” “rarely,” “sometimes,” “mostly,” and “always”) regarding the respective items: “I did less than I wanted” and “I was only able to do certain things.” Another item assessed the response on a 5-point Likert-type scale (ranging from *not at all* to *very much*) to the question: “To what extent has pain prevented you from performing everyday activities at home and at work in the past 4 weeks?”. The last item assessed the answer on a 5-point Likert-type scale (ranging from *never* to *always*) to the question “How often has your physical health impaired your contact with other people (visits to friends, relatives, etc.) in the past 4 weeks?” All six items were combined in one variable assessing *physical constraint*. Internal consistency was good, α = 0.85.

#### Risk Awareness

We assessed the additional variable risk awareness which defines the quality and quantity of a person’s susceptibility to a specific health threat. We considered risk awareness to be related to physical constraint and general health. We used three items from the risk awareness questionnaire by Schwarzer et al. (2003) and added one item that was developed by a research group of our department. The short questionnaire started with the following introduction “If I compare myself to an average person of my age and gender, then … is …” and the respective items could be answered by five answer options (“far below the average,” “below the average,” “just like the average,” “above the average,” and “far above the average”): “my risk of getting chronic pain at some point,” “my risk of having restricted mobility at some point,” “my risk of getting a very serious illness 1 day,” and “my physical fitness (reversed item).” Internal consistency was acceptable, α = 0.75.

#### General Health

To measure general health, we used one additional item from the SF-12 (Ware et al., 1996). Participants indicated on a 5-point Likert-type scale (ranging from *bad* to *excellent*) how they would describe their state of health in general.

### Design and Procedure

First, participants answered a short demographic questionnaire assessing age, gender, and years of education. Afterward, they completed the statements measuring general health, physical constraint, and risk awareness. Then, participants read the typical instructions of the classical MMG (Sokolowski et al., 2000) before they rated the 42 picture scenarios (28 pictures for the MMG-M plus 14 scenarios from the classical MMG) regarding the motive statements. The picture scenarios were presented in random order to prevent any transfer effects. Each picture scenario was shown in the upper central part of the screen. Below each picture, the 12 statements from the MMG were presented. Participants could agree or disagree with the respective statements. After processing the statements, participants rated three additional items which assessed how much the presented scenario referred to mobility, activity, or health (also presented in random order). The mobility item served as a selection criterion deciding the final selection of 14 pictures of the stimulus material for the MMG-M. Because we perceived the mobility rating as a rather explicit way to assess the mobility perception of participants in each scenario, we furthermore conducted a short response time task, showing the 42 picture scenarios again in random order. Participants were instructed to indicate as spontaneously as possible whether the shown scenario represented a mobility situation or not. The results from this short response time task for the selected pictures can be found in Table 2. Results from the mobility ratings and the response time task were largely identical.

In the follow-up survey, participants also answered a short demographic questionnaire assessing age, gender, and years of education. Then, they read the following instruction (translated from German) that was adapted from Sokolowski et al. (2000): “The following study will be about the perception of images. All people find themselves in different, constantly changing life situations every day. With the change of the situations naturally also thoughts and feelings change. As is well known, there are also great differences between people. This is about how well you can put yourself in social situations. Each picture situation is supposed to represent an everyday life situation. The pictures are deliberately shown a bit ambiguously. Therefore, try to let your imagination run free and put yourself in the role of any person in the picture. Under each picture situation you are asked to answer three questions. Please rate each picture in terms of how much it represents the themes of achievement, affiliation, or power to you.” Afterward, the 28 newly created picture scenarios were presented with the three questions (“How much does the above situation illustrate an achievement/affiliation/power theme?”). Again, the picture scenarios as well as the three questions were presented in random order to prevent any transfer effects from occurring.

**FIGURE 2:**
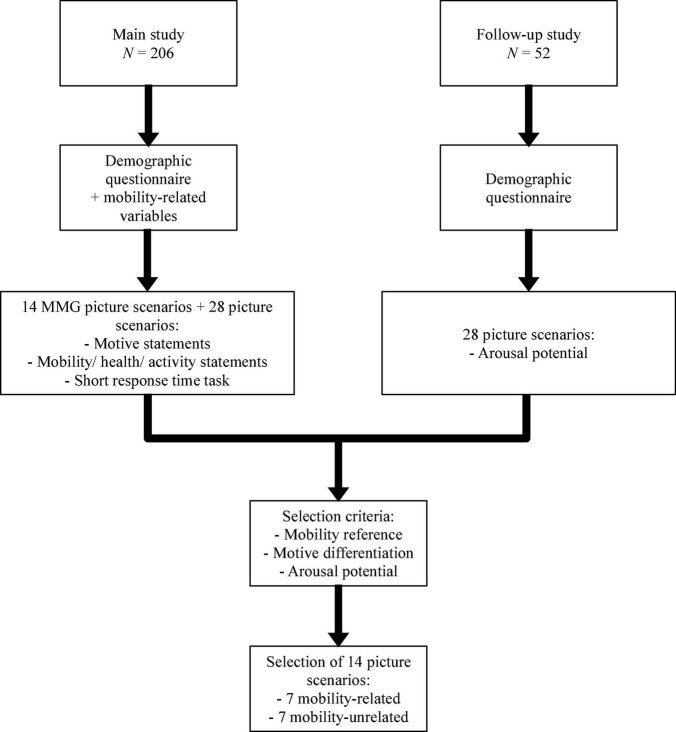
Flow figure visualizing the study design of the main and follow-up survey.

A flow figure presenting the study procedure of the main and follow-up survey is depicted in Figure 2.

### Statistical Analyses

In addition to conducting correlational analyses, reliability analyses, and mean comparisons (*t*-tests) between the mobility and non-mobility situations, we performed confirmatory factor analyses in order to test the model fit of the newly developed MMG-M. We used the *lavaan* package (Rosseel, 2012) from the statistical computing framework R (R Core Team, 2020) with maximum likelihood estimation. We conducted confirmatory factor analyses for a three-factor model (comprising the factors achievement motive, affiliation motive, and power motive) with and without mobility factor, for a two-factor model (hope and fear component) with and without mobility factor as well as for a six-factor model (the three motives including the hope and fear components), again with and without mobility factor. Moreover, we compared the MMG-M models with the classical MMG. Based on the study by Sokolowski et al. (2000), we also added up the raw scores across the picture scenarios. For the MMG-M models, we combined the two items for each motive component (e.g., the two items measuring fear of rejection) in order to reduce model complexity (e.g., Bandalos, 2002; Matsunaga, 2008) and to achieve the same number of items for the MMG and MMG-M models^2^. We used four criteria to evaluate the model fit: a chi-square significance test, the Root Mean Squared Error of Approximation (*RMSEA*), the Comparative Fit Index (*CFI*), and the Standardized Root Mean Residual (*SRMR*). We used the quasi-standard cut-offs suggested by Hu and Bentler (1999).

## Results

### Determination of the Factor Structure of the Multi-Motive Grid Mobility

Confirmatory factor analyses were conducted to analyze whether the data of the MMG and MMG-M were best fit by a three-factor (achievement, affiliation, and power motive), a two-factor (hope and fear component), or a six-factor model (the three motives including the hope and fear components). Moreover, for the MMG-M, we investigated whether the additional inclusion of a mobility factor improved the model fit. The latent covariance between the factors was included in each model. The results of the respective factor models are presented in Table 3.

**TABLE 3:** Model fit statistics for factor models of the MMG and MMG-M.

Model	χ^2^	χ^2^/*df*	*CFI*	*SRMR*	*RMSEA*
**Three-factor model**					
MMG	396.50	7.77	0.76	0.10	0.18
MMG-M	636.81	12.49	0.62	0.14	0.24
**Three-factor model with mobility factor**					
MMG-M	461.00	10.24	0.73	0.14	0.21
**Two-factor model**					
MMG	209.26	3.95	0.89	0.07	0.12
MMG-M	296.41	5.59	0.84	0.07	0.15
**Two-factor model with mobility factor**					
MMG-M	228.35	4.86	0.88	0.07	0.14
**Six-factor model**					
MMG	91.78	2.35	0.96	0.05	0.08
MMG-M	189.50	4.86	0.90	0.05	0.14
**Six-factor model with mobility factor**					
MMG-M	85.20	2.58	0.97	0.04	0.09

*RMSEA, Root Mean Squared Error of Approximation; CFI, Comparative Fit Index; SRMR, Standardized Root Mean Residual.*

**FIGURE 3:**
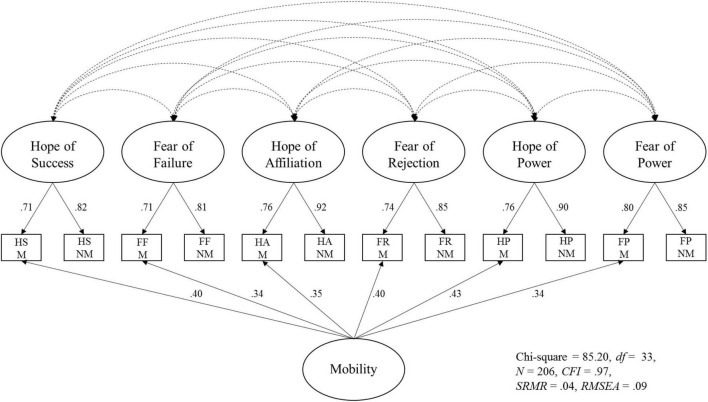
Visualization of the factor model comprising the six main latent factors plus an additional latent factor for the mobility-related items (M, mobility; NM, non-mobility). Covariance between the main latent factors is included in the model, but the specific values are not presented in this figure. Residual variance of the respective items is also included in the model but not shown in this figure.

The global fit of the three-factor models was not acceptable, *CFI* was <0.95, while *SRMR* and *RMSEA* were both >0.08. The global fit of the two-factor models was also not acceptable, *CFI* was <0.95 and *RMSEA* > 0.08. However, the model fit of the six-factor model was good for the MMG, *CFI* was >0.95, while *SRMR* and *RMSEA* were both ≤0.08. For the MMG-M, the six-factor model including an additional mobility factor also showed a good model fit, *CFI* was >0.95, *SRMR* was <0.05, and *RMSEA* was <0.09. When comparing the model fit of the six-factor model including the mobility factor with the model fit of the six-factor model without mobility factor regarding the MMG-M, the difference between the chi-square values was significant, χ^2^_*difference*_(6) = 104.30, *p* < 0.001, indicating that the six-factor model including the mobility factor is preferable. The six-factor model with additional mobility factor for the MMG-M is shown in Figure 3.

### Interscale Correlations of the Six Motive Components

The Pearson product-moment correlations among the six motive components [hope of success (HS), hope of affiliation (HA), hope of power (HP), fear of failure (FF), fear of rejection (FR), and fear of power (FP)] – separately for the MMG and MMG-M – are presented in Table 4. All motives were significantly correlated, all *p* < 0.001. The correlation coefficients were largely identical when descriptively comparing them between the classical MMG and the newly developed MMG-M. For the MMG, the range of correlation coefficients between the hope components ranged from *r* = 0.61 to *r* = 0.80, and between the fear components from *r* = 0.60 to *r* = 0.72. Regarding the intercorrelations between hope and fear components, the correlation coefficients ranged from *r* = 0.31 to *r* = 0.67. For the MMG-M, the pattern was very similar. The range of correlation coefficients between the hope components ranged from *r* = 0.65 to *r* = 0.77, and between the fear components from *r* = 0.63 to *r* = 0.79. Regarding the associations between hope and fear components, the correlation coefficients ranged from *r* = 0.28 to *r* = 0.57. Table 4 shows that the associations within the hope and fear components were stronger compared to the association across fear and hope components.

**TABLE 4:** Pearson product-moment correlations among the six motives measured by the MMG and by the MMG-M.

Motive	HS	HA	HP	FF	FR	FP
**MMG**						
HS		0.69***	0.80***	0.41***	0.44***	0.52***
HA			0.61***	0.39***	0.46***	0.31***
HP				0.47***	0.55***	0.67***
FF					0.63***	0.60***
FR						0.72***
**MMG-M**						
HS		0.74***	0.77***	0.42***	0.34***	0.38***
HA			0.65***	0.28***	0.35***	0.26***
HP				0.47***	0.49***	0.57***
FF					0.63***	0.63***
FR						0.79***

***p < 0.001. HS, hope of success; HA, hope of affiliation; HP, hope of power; FF, fear of failure; FR, fear of rejection; FP, fear of power.

### Calculation of Internal Consistencies of the Motive Components

We estimated the internal consistencies by conducting Cronbach’s Alpha for the respective items across all presented picture scenarios. The alpha coefficients as well as mean and standard deviation of each motive component are shown in Table 5. Internal consistencies for the six motive scores of the MMG had a range from α = 0.81 to α = 0.85, and from α = 0.82 to α = 0.88 for the MMG-M. Internal consistencies of the MMG and MMG-M motive components were therefore highly comparable. Moreover, we estimated internal consistencies separately for the mobility-related and mobility-unrelated motive components of the MMG-M. For mobility-related motives, the alpha coefficients ranged from α = 0.74 to α = 0.83, whereas a range of α = 0.72 to α = 0.80 was observed for the mobility-unrelated motive components. The complete results can be found in Table 6, together with the mean and standard deviations of the six motive scores.

**TABLE 5:** Mean (*M*), standard deviation (*SD*), and internal consistency (α) of the six motive scores of the MMG and the MMG-M.

Motive Score	*M*	*SD*	α
**HS**			
MMG	17.41	5.08	0.83
MMG-M	16.80	6.14	0.88
**HA**			
MMG	13.24	5.14	0.82
MMG-M	16.65	5.90	0.87
**HP**			
MMG	17.38	5.80	0.85
MMG-M	15.65	6.08	0.86
**FF**			
MMG	10.73	5.23	0.81
MMG-M	10.04	5.23	0.82
**FR**			
MMG	11.66	5.15	0.82
MMG-M	10.26	5.74	0.86
**FP**			
MMG	14.82	5.43	0.84
MMG-M	10.48	5.77	0.86

*HS, hope of success; HA, hope of affiliation; HP, hope of power; FF, fear of failure; FR, fear of rejection; FP, fear of power.*

**TABLE 6:** Mean (*M*), standard deviation (*SD*), and internal consistency (α) of the six motive scores of the MMG-M, separated by mobility and non-mobility situations. *T*-values and effect sizes (*d*) are reported to indicate differences in motive scores between mobility and non-mobility situations.

Motive score	*M*	*SD*	α	*t-value*	*d*
**HS**					
Mobility	9.30	3.54	0.83	8.07***	0.56
Non-mobility	7.50	3.39	0.80		
**HA**					
Mobility	8.07	3.21	0.79	2.98*	0.21
Non-mobility	8.58	3.18	0.77		
**HP**					
Mobility	7.44	3.55	0.81	3.95***	0.28
Non-mobility	8.20	3.12	0.74		
**FF**					
Mobility	6.09	3.08	0.74	10.88***	0.76
Non-mobility	3.95	2.85	0.72		
**FR**					
Mobility	4.51	3.27	0.79	6.32***	0.44
Non-mobility	5.74	3.11	0.77		
**FP**					
Mobility	4.96	3.30	0.78	3.06*	0.21
Non-mobility	5.51	3.02	0.75		

***p < 0.001, *p < 0.05. HS, hope of success; HA, hope of affiliation; HP, hope of power; FF, fear of failure; FR, fear of rejection; FP, fear of power.

### Differences Between Motives in Mobility and Non-mobility Scenarios

Independent *t*-tests were conducted to investigate whether the scores of the motive components differed between mobility and non-mobility situations. Because the tests represented families of related tests, we used Bonferroni correction to prevent alpha-error accumulation. The results are depicted in Table 6. Hope of success and fear of failure were significantly higher in mobility compared to non-mobility situations, *t*(205) = 8.07, *p* < 0.001, *d* = 0.56, 95% *CI* [0.35, 0.75] and, *t*(205) = 10.88, *p* < 0.001, *d* = 0.76, 95% *CI* [0.53, 0.93], respectively. Hope of affiliation and fear of rejection were significantly higher in non-mobility compared to mobility scenarios, *t*(205) = 2.98, *p* = 0.019, *d* = 0.21, 95% *CI* [0.01, 0.40] and, *t*(205) = 6.32, *p* < 0.001, *d* = 0.44, 95% *CI* [0.24, 0.63], respectively. Hope of power and fear of power were also significantly higher in non-mobility compared to mobility situations, *t*(205) = 3.95, *p* < 0.001, *d* = 0.28, 95% *CI* [0.07, 0.45] and, *t*(205) = 3.06, *p* = 0.015, *d* = 0.21, 95% *CI* [0.01, 0.40], respectively.

### Associations With Age, Gender, General Health, Physical Constraint, and Risk Awareness

The Pearson product-moment correlations between the motive scores of the mobility and non-mobility scenarios with age, gender, general health, physical constraint, and risk awareness are presented in Table 7. There were significant positive associations between the hope components of the achievement and affiliation motive and age for both mobility and non-mobility situations (ranging from *r* = 0.20 to *r* = 0.25, all *p* < 0.003). Moreover, age was negatively correlated with fear of rejection in non-mobility situations (*r* = −0.31, *p* < 0.001), and was also negatively associated with fear of power in mobility and non-mobility scenarios (*r* = −0.16, *p* = 0.026, and *r* = −0.24, *p* < 0.001, respectively).

**TABLE 7:** Correlations of the six motive scores for mobility and non-mobility scenarios as well as of mobility perception in mobility and non-mobility situations with age, gender*^a,b,c^*, general health (GH), physical constraint (PhC), and risk awareness (RA).

Motive score	Age	Gender	GH	PhC	RA
**HS**					
Mobility	0.25**	–0.06	–0.09	–0.06	0.05
Non-mobility	0.21**	0.03	−0.22**	0.13	0.09
**HA**					
Mobility	0.22**	–0.01	–0.05	–0.04	–0.02
Non-mobility	0.20**	0.05	−0.15*	0.08	0.10
**HP**					
Mobility	0.09	–0.09	–0.04	–0.02	0.01
Non-mobility	0.06	–0.07	–0.13	–0.01	0.04
**FF**					
Mobility	–0.05	−0.27**	–0.09	0.10	0.25**
Non-mobility	–0.12	–0.01	–0.11	0.21**	0.20**
**FR**					
Mobility	–0.07	–0.07	–0.03	0.19**	0.21**
Non-mobility	−0.31**	−0.19**	0.04	0.06	0.22**
**FP**					
Mobility	−0.16*	–0.12	–0.05	0.15*	0.20**
Non-mobility	−0.24**	–0.04	–0.04	0.14	0.19**
**Mobility perception**					
Mobility	0.00	–0.14	0.01	−0.15*	–0.02
Non-mobility	0.20**	0.05	–0.13	0.18**	0.03

*p < 0.05, **p < 0.01. HS, hope of success; HA, hope of affiliation; HP, hope of power; FF, fear of failure; FR, fear of rejection; FP, fear of power.

*^a^N = 201 (participants with diverse gender were not included).*

*^b^1 = female, 2 = male.*

*^c^Spearman’s rank-order correlation is reported.*

Gender was negatively related to fear of failure in mobility situations (*r* = −0.27, *p* < 0.001) and negatively correlated with fear of rejection in non-mobility scenarios (*r* = −0.19, *p* = 0.007). Accordingly, men stated lower levels of fear of failure in mobility situations and lower levels of fear of rejection in non-mobility scenarios compared to women.

General health was negatively associated with hope of success (*r* = −0.21, *p* = 0.001) and hope of affiliation (*r* = −0.19, *p* = 0.019) in non-mobility situations. Most interestingly, physical constraint was positively related to fear of rejection (*r* = 0.19, *p* = 0.005) and fear of power (*r* = 0.15, *p* = 0.032) in mobility situations. It was also positively associated with fear of failure in non-mobility scenarios (*r* = 0.21, *p* = 0.001). Lastly, there were significant and positive associations between all fear components and risk awareness for both mobility and non-mobility situations (ranging from *r* = 0.19 to *r* = 0.25, all *p* < 0.006).^3^

We also examined the association between mobility perception in mobility and non-mobility situations with age, gender, general health, physical constraint, and risk awareness. There were significant and positive associations between mobility ratings and age (*r* = 0.20, *p* = 0.002) as well as between mobility ratings and physical constraint in non-mobility scenarios (*r* = 0.18, *p* = 0.005), indicating a higher mobility perception in non-mobility situations for older adults and individuals with higher physical restrictions. Lastly, there was a significant negative correlation between mobility ratings and physical constraint regarding mobility scenarios (*r* = −0.15, *p* = 0.028).

## Discussion

Previous investigations mainly focused on either categorizing reasons to be mobile or on barriers that prevent high mobility in older adults or individuals with physical restrictions (Rasinaho et al., 2007; Gutiérrez et al., 2018; Rodrigues et al., 2019). In the current study, we developed an assessment to measure three fundamental psychological motives – achievement, affiliation, and power – and their corresponding hope and fear components in mobility-related and mobility-unrelated scenarios. Based on the grid technique by Sokolowski et al. (2000), which aims to assess all three motives in one measure, we created the MMG-M assuming that psychological motives might be activated to a different extent in mobility and non-mobility scenarios especially for individuals with physical limitations. We selected a broad spectrum of pictures for the mobility and non-mobility scenarios that allowed a good differentiation in the underlying motive components. An essential distribution in the investigated psychological motives is necessary especially when aiming to reveal associations between the motives with other constructs of interest or in order to predict behavior in future investigations using the MMG-M.

By means of confirmatory factor analyses, we revealed a good model fit for the newly developed MMG-M defining six latent factors (hope of success, hope of affiliation, hope of power, fear of failure, fear of rejection, and fear of power) and an additional mobility factor. The model including this mobility factor had a significantly better model fit compared to the model without mobility factor. Moreover, the model fit was highly comparable to the fit of the classical MMG by Sokolowski et al. (2000). Therefore, the underlying motive structure comprising six factors is also given in the MMG-M. Moreover, the better model fit for the six-factor solution including the mobility factor further underlines the importance of the differentiation between mobility and non-mobility-related motive components. Furthermore, the intercorrelations within and between the hope and fear components were largely comparable with those of the classical MMG (Sokolowski et al., 2000). Internal consistencies were very similar when comparing the MMG-M with the MMG, indicating good reliability when assessing the motive components in the newly developed mobility and non-mobility scenarios.

When contrasting the motive scores between mobility-related and mobility-unrelated picture scenarios, we identified essential differences. Whereas both components of the achievement motive were pronounced more strongly in mobility situations, both affiliation and power motive components were higher in non-mobility scenarios. It is possible that the physical character of mobility (e.g., movement and physical activity) activates the achievement motive to a stronger degree. In non-mobility situations, the interpersonal interaction might be more pronounced and therefore addresses the affiliation and power motive more strongly.

More interesting than examining differences between the two scenario types is the question of which individual differences might be associated with the respective motive characteristics in mobility and non-mobility situations. We found risk awareness to be related to all three fear components in both mobility-related and mobility-unrelated scenarios. This fits with a common finding that anxious individuals overestimate the risk that negative events might happen in their lives and that they have a more pessimistic view compared to people with lower anxiety levels (Kim et al., 2020). We were also able to identify essential relations between gender and fear of rejection in the non-mobility situations as well as between gender and fear of failure in mobility scenarios. The first finding is in line with previous empirical evidence suggesting that women score higher in implicit affiliation motivation (Drescher and Schultheiss, 2016). In the case of the second finding regarding the achievement motive, no gender differences were reported in the mentioned meta-analysis (Drescher and Schultheiss, 2016), but there is some evidence that men score higher regarding the achievement motive compared to women (Denzinger et al., 2016). As elaborated earlier, the achievement motive components were activated more strongly in mobility scenarios maybe due to the stronger physical character of the presented situations. It might be possible that gender differences in achievement motivation are more prevalent in mobility-related situations because of their higher physical association.

The most interesting associations could be found for physical constraint and age. Physical constraint was positively related to fear of rejection and fear of power in mobility scenarios. We assume that individuals with higher physical constraint feel less in control regarding mobility situations (component “fear of power”) and might fear being rejected by other individuals because they cannot keep up with them (component “fear of rejection”). This also fits the finding that older and more physically restricted individuals perceived more mobility in non-mobility situations compared to younger and physically less restricted adults, indicating that some characteristics of the presented scenarios might become more mobility-related as one gets older or experiences some sort of mobility impairment. These findings underline the importance of considering mobility-related scenarios when investigating underlying psychological motive dispositions.

It is unclear why higher levels of physical constraint were negatively associated with mobility perception in mobility scenarios. One explanation might be that for people with higher physical constraints, mobility is relevant in many everyday situations and that there are less differences between situations. This would explain why they were more sensitive for mobility in non-mobility situations and less sensitive for mobility in more mobility-related situations. However, given the difference regarding the associations of physical constraint especially with fear of rejection in mobility-related and non-mobility situations, individuals with stronger physical restrictions still seem to differ in their perception of those scenarios.

Lastly, most of the significant associations could be found between age and the respective motives, which highlights the necessity to recruit age-heterogeneous samples when creating new assessment tools measuring implicit motives especially when contrasting mobility and non-mobility situations. Whereas age was positively correlated to hope of success and hope of affiliation as well as negatively related to fear of power in both mobility and non-mobility scenarios, there was an essential difference regarding the motive component fear of rejection. There was only a significant and negative correlation for non-mobility situations. According to the socioemotional selectivity hypothesis (Carstensen, 1992), emotion-related goals become more salient and important compared to knowledge-focused goals during the life span. Older people tend to spend more time with emotionally meaningful partners whereas young individuals engage in making new contacts (Fredrickson and Carstensen, 1990; Fung et al., 1999). We believe that older participants have a smaller but stable social network compared to younger adults where social rejection is less likely to occur in comparison to situations, in which new contacts will be made. This is reflected in our results by a negative correlation between age and fear of rejection in non-mobility situations in which fear of rejection was generally pronounced more strongly compared to mobility scenarios.

### Limitations and Future Directions

In our study, we tested the reliability of our newly-developed MMG-M by means of internal consistency which represents the homogeneity of the respective items across picture scenarios. However, this does not indicate the retest correlation when assessing the MMG-M items with a time interval. Even though we believe that the retest reliability should be in the same range compared with the study of Sokolowski et al. (2000) because we used the same 12 statements for each picture scenario, future investigations should examine this point in more detail.

In addition, the external validity of the MMG-M should be investigated more thoroughly in future studies. Like in the study by Puca and Schmalt (1999), it could be tested whether approach and avoidance orientation are associated with a more pronounced hope or fear component, respectively. The authors found that approach-oriented individuals showed higher hope compared to fear scores regarding the achievement motive. Avoidance-oriented participants – on the other hand – scored higher with regard to the fear component. In a diary study, it was found that affiliation themes that occurred in individuals’ daily lives were significantly predicted by affiliation motivation (Sokolowski et al., 2000). In addition, Sokolowski and Kehr (1999) investigated industrial managers in a training program and revealed that only the power motive was able to predict their gain in leadership competence during the program.

For our MMG-M, it would be further interesting to test the external validity of the six motive components in terms of mobility-related contexts, for example by investigating participants in rehabilitation facilities or of higher frailty levels in general. Since our sample represented a rather healthy sample with a relatively small range of physical limitations, it would be fruitful to investigate individuals with a broader spectrum of physical constraints. Although we could find that people with stronger physical limitations showed higher fear of rejection and fear of power in mobility situations, these associations should be even more pronounced in individuals with higher frailty levels. In addition to the focus on determinants of motive structures, future studies should investigate the link between motives in mobility and non-mobility situations, and actual behavior. The association between motives and behavior is at the core of motivational theories and thus deserves special attention when developing a motive measure. In our present study, the novelty lies in the assumption of motive structures specific to situational classes. Therefore, we were mainly interested in showing which factors contribute to differences in motive structures across situational classes. Having established the fact of situational class-dependent motive structures, the logical next step would be to test consequences of these differences on a behavioral level.

Nevertheless, the current study provides a first insight that the fear components for the affiliation and power motive are more distinct in mobility situations for individuals with mobility impairments. This is an important finding because it implicates that physically limited individuals might associate mobility situations with the fear of being rejected or of not being in control in the respective situation. This also raises the question about which interventions are necessary and which infrastructure and support systems are essential to reduce these concerns. Maintaining a high social participation in society plays an important role to prevent deterioration in well-being and to preserve a high quality of life (Metz, 2000; Groessl et al., 2007; Yeom et al., 2008).

Lastly, the current study suggests that, despite the assumed transsituationality of motives, it might prove useful to investigate the context-dependence of different motives and how this can be understood in a lifespan perspective on human development.

## Data Availability Statement

The dataset presented in this study can be found in online repositories. The data for this study is available at https://osf.io/3u5fc/.

## Ethics Statement

The studies reported in this article were conducted in accordance with the Declaration of Helsinki and the national ethical guidelines of the authors’ university, where the data was collected. The patients/participants provided their written informed consent to participate in this study.

## Author Contributions

AM contributed to the conceptualization, data collection, data preparation, data analysis, and writing – original draft preparation. MT contributed to the conceptualization, data analysis, and writing – review and editing. JF contributed to the supervision, conceptualization, and writing – review and editing. All authors contributed to the article and approved the submitted version.

## Conflict of Interest

The authors declare that the research was conducted in the absence of any commercial or financial relationships that could be construed as a potential conflict of interest.

## Publisher’s Note

All claims expressed in this article are solely those of the authors and do not necessarily represent those of their affiliated organizations, or those of the publisher, the editors and the reviewers. Any product that may be evaluated in this article, or claim that may be made by its manufacturer, is not guaranteed or endorsed by the publisher.

## Footnotes

1 Note that it was not always possible to achieve this criterion perfectly. However, when combining the item difficulties for the items across the picture scenarios, the item difficulties met the criterion well.

2 Note that for the MMG-M models each motive component is represented twice (for mobility and non-mobility scenarios).

3 To account for the overlap between the six motive components, we conducted additional regression analyses separately for general health, risk awareness, and physical constraint as well as for the mobility-related and mobility-unrelated motive components. For general health, no regression coefficient reached significance, all *p* > 0.07. In case of risk awareness as dependent variable, mobility-related fear of failure, β = 0.20, *p* = 0.024, and mobility-unrelated hope of power, β = −0.23, *p* = 0.035, reached significance. Lastly, for physical constraint, mobility-related fear of rejection was marginally significant, β = 0.21, *p* = 0.054, and mobility-unrelated fear of failure, β = 0.20, *p* = 0.025, as well as hope of power, β = −0.29, *p* = 0.008, reached significance. However, given the substantial overlap between the motive components, these results must be interpreted with caution due to multicollinearity issues.
